# Effects of substitution level and particle size of extruded soybean hull fractions on physicochemical and sensorial properties of high‐fiber pan bread during storage

**DOI:** 10.1002/fsn3.3027

**Published:** 2022-10-06

**Authors:** Forough Sadat Tabibloghmany, Mostafa Mazaheri Tehrani, Arash Koocheki

**Affiliations:** ^1^ Department of Food Science and Technology Ferdowsi University of Mashhad (FUM) Mashhad Iran

**Keywords:** extrusion, pan bread, particle size, physicochemical properties, soybean hull

## Abstract

The effect of adding different fractions of extruded and non‐extruded soybean hull to wheat flour at 20% and 30% and two‐particle size levels (smaller and larger than 150 μm) was studied on the physicochemical, sensorial properties, and the shelf‐life of high‐fiber molded bread. Increasing the amount of all different fractions of the soybean hull raised the water absorption of the dough. It also increased the ash and crude fiber contents, bread crust lightness, redness and yellowness, bread crumb hardness as well as the cells number per unit area of the crumb. Moreover, it reduced the moisture content, specific volume, porosity, and overall acceptability of the pan bread. The treatments containing the fractions with larger particle sizes of the soybean hull had higher dough stability time, bread‐specific volume, porosity, and lightness, as well as lower crumb hardness and moisture content, and crust redness and yellowness than the corresponding ones with finer particle sizes. The samples prepared with the extruded fractions with smaller particle sizes showed lower moisture content, hardness, porosity, and specific volume. After studying the bread staling, moisture content and overall acceptance of the samples decreased. In addition, the enthalpy in differential scanning calorimetry **(**DSC) and the signal intensity in x‐ray diffraction (XRD) increased during storage. In many cases, the bread with the large‐sized extruded fractions of soybean hull at the substitution level of 20% was the most suitable product in most of the variables studied.

## Graphical abstract

we could produce high dietary fiber pan bread (20%) contain large‐sized soybean hull fractions. It had acceptable texture properties, overall acceptance, and became less stale over time than corresponding similar bread.

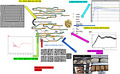

## 1 INTRODUCTION

Bread is one of the most widely consumed foods in the world. Its annual average consumption rate is of 41–303 kg per person (Rosell, 2011). According to the available data, more than half of the European Union population (52%) is overweight or obese (WHO, 2010). For this reason, it is essential to produce suitable bread for people with metabolic disorders like diabetes (López et al., 2004).

Although, wheat bran is utilized as a cheap and rich source of fiber in dietary bread, it is problematic to use it in large quantities and with different particle sizes. There is a decrease in bread volume because of a reduction in the dough gas storage capacity at high substitution levels. The weakening of the gluten network and the interaction between the fiber and the protein network are the reasons for this reduction (Noort et al., 2010). In the literature, the reported results regarding the effect of wheat bran particle size on the final quality of bread are different and even contradictory (López et al., 2004; Majzoobi et al., 2014; Noort et al., 2010; Rosell, 2011). Unlike wheat bran, which is problematic to be ground finely and produces fractions with different sizes and physicochemical and nutritional properties—soybean hull is much easier to be ground. The grounded soybean hull has a uniform particle size distribution (Ayo & Kajo, 2016; Sarfaraz et al., 2018). One of the nutritional advantages of soybean hull over wheat bran is retaining the bioavailability of divalent elements, as the amount of phytate (anti‐nutritional factor) in the former is much smaller than in the latter (Ayo & Kajo, 2016). The higher total amount of dietary fiber, iron, and calcium contents in soybean hull has made it very appropriate for bread fortification in comparison with wheat bran (Chee et al., 2005; Johnson et al., 1985).

It has been shown that it is possible to use more soluble dietary fiber (SDF) in bakery products due to its better technological and functional characteristics and higher fermentability than insoluble dietary fiber (IDF; Rao et al., 2007).

Extrusion cooking is a process that causes the disruption of cellulosic polymers in the cell wall structure. This process increases SDF by applying heat, pressure, and high shear forces to the soybean hull. Optimization of the soybean hull extrusion brings about a significant rise in SDF compared with non‐extruded one. The higher water absorption and solubility indices in the extruded soybean hull in comparison with the non‐extruded one show its greater potential for producing products which are freshly stored and require hydration, including bakery products and frozen foods (Tabibloghmany et al., 2020).

Mahasowanwong (1997) studied the formulation of fiber‐enriched soybean hull bread. The physical properties of the bread containing10%–15% (w/w) soybean hull were investigated in three levels of the hull particle size: coarse (mesh larger than 100; pore size more than 150 microns), medium (mesh between 100 and 60; pore size between 250 and 150 microns), and fine (mesh smaller than 100; pore size less than 150 microns). Preliminary sensory evaluation showed that bread with 10% large‐sized soybean hull was preferred (Mahasowanwong, 1997).

Other researchers added soybean hull to the flour for preparing chapatti bread (non‐leavened flatbread) at 1.5%, 3%, 4.5%, 6%, and 7.5%. The composite flour and chapatti bread were analyzed in chemical, rheological, and baking properties. The effect of the soybean hull was significant (*p* < .05) on water absorption during dough development and stability times. Increasing the amount of soybean hull improved the nutritional properties of the bread. But the most acceptance of sensory characteristics (color, flavor, foldability, and chewability) was seen at 4.5% (Khan et al., 2005).

Ayo and Kajo (2016) investigated the effects of soybean hull on the properties of acha grains‐based biscuits. The soybean hull flour was substituted for the acha flour at 0%, 2.5%, 5.0%, and 7.5% to produce soybean hull – acha composite flour used in the biscuit production. With increasing the levels of soybean hull, the contents of fiber, ash, fat, and protein increased, while the carbohydrates content decreased. Moreover, the average scores of color and taste decreased, whereas crispness, odor, and texture increased. In total, the product with 2.5% soybean hull showed the highest sensory acceptance (Ayo & Kajo, 2016).

Despite the use of soybean hulls in many bakery products, no research has so far been done about the effects of the amounts exceeding 15% and the particle sizes of different fractions of extruded and non‐extruded soybean hull on the physicochemical properties and shelf‐life of high‐fiber pan bread. Therefore, to provide dietary fiber and minerals, to create variety in the bread consumed, and to study new structures, the use of various fiber sources such as different fractions of soybean hull in bread formulations is suggested. Although so far, the use of different fractions of extruded soybean hull at concentrations less than 15% had not been studied in the wheat dough formulation, the bread produced at these concentrations had no significant difference with non‐extruded fractions. At replacement levels of less than 15%, both extruded and native soybean hull fractions created bread with acceptable quality (data not shown). Therefore, the subject of this research was determined to study bread with a percentage of more than 15% of soybean hull.

## 2 MATERIALS AND METHODS

### 2.1 Materials

Soybean hull (*DTX variety* containing about 8%–10% soybean) was purchased from Toos Soya Protein Co. (Mashhad, Iran). White wheat flour (77% extraction rate, 0.5% ash, 10.97% protein, 0.92% fat, and 8.4% humidity) and wheat bran were purchased from Neyshabur Flour Co. (Mashhad, Iran). Instant dry yeast (Razavi Co., Mashhad, Iran), improver (Shabnam‐Pouyesh Co., Mashhad, Iran), salt, sugar, and sunflower oil were supplied from the local market. The KTDFR‐200A enzyme kit was gifted by Megazyme (Bray, Ireland). All the chemicals used in this research were of analytical grade.

### 2.2 Methods

#### 2.2.1 Preparation of soybean hull

The soybean hull was milled (Ball mill, Toos shekan, Khorasan, Iran) and passed through a screen with a mesh number of 2. Then, it was (manually) mixed with distilled water to reach the optimal moisture level (40%). Subsequently, the prepared soybean hull was agitated (manually) for 10 min so that the humidity to be spread completely. It was then packaged in polyethylene bags and vacuum‐sealed (vacuum sealer, JauKang, China). Next, the soybean hull was allowed to equilibrate its moisture content overnight at 4°C before extrusion, which was done using a co‐rotating twin‐screw extruder (DS56, Jinan Saxin, China) with a length: diameter (L:D) ratio of 10:1 and a die diameter of 5 mm.

According to the results of the extrusion process optimization, the screw rotational speed, feed moisture content, and temperature of the extruder chamber were set at 180 rpm, 40%, and 85°C, respectively (Tabibloghmany et al., 2020). The extruded soybean hull was dried in a vacuum oven (vacuum oven drying VACIOTEM, Ukraine) at 40°C to reach a moisture content below 7% (wet basis). Afterward, it was ground and passed through a sieve, 500 μm in pore size. Also, with the same grinder (Ball mill, Toos shekan, Khorasan, Iran) and sieve (500 μm in pore size), the non‐extruded soybean hull was prepared.

A sieve with a mesh size of 100 (150 μm in pore size) was applied to separate the different fractions of the soybean hull. In this research, the particles on and under the sieve were the sifted and refusal particles, respectively.

#### 2.2.2 Preparation of dough and pan bread

The bread formulation consisted of 20% or 30% of the different fractions of both extruded (EB20, ES20, EB30, and ES30) and non‐extruded soybean hull with different particle sizes (BB20, BS20, BB30, and BS30). A sample containing 20% wheat bran instead of soybean hull, was prepared as the control (W20). The amounts of 1.5% salt, 1.5% sugar, 1.8% active dry yeast, 0.5% alpha‐amylase improver, and 5% sunflower oil were the same in all the samples.

**FIGURE 1:**
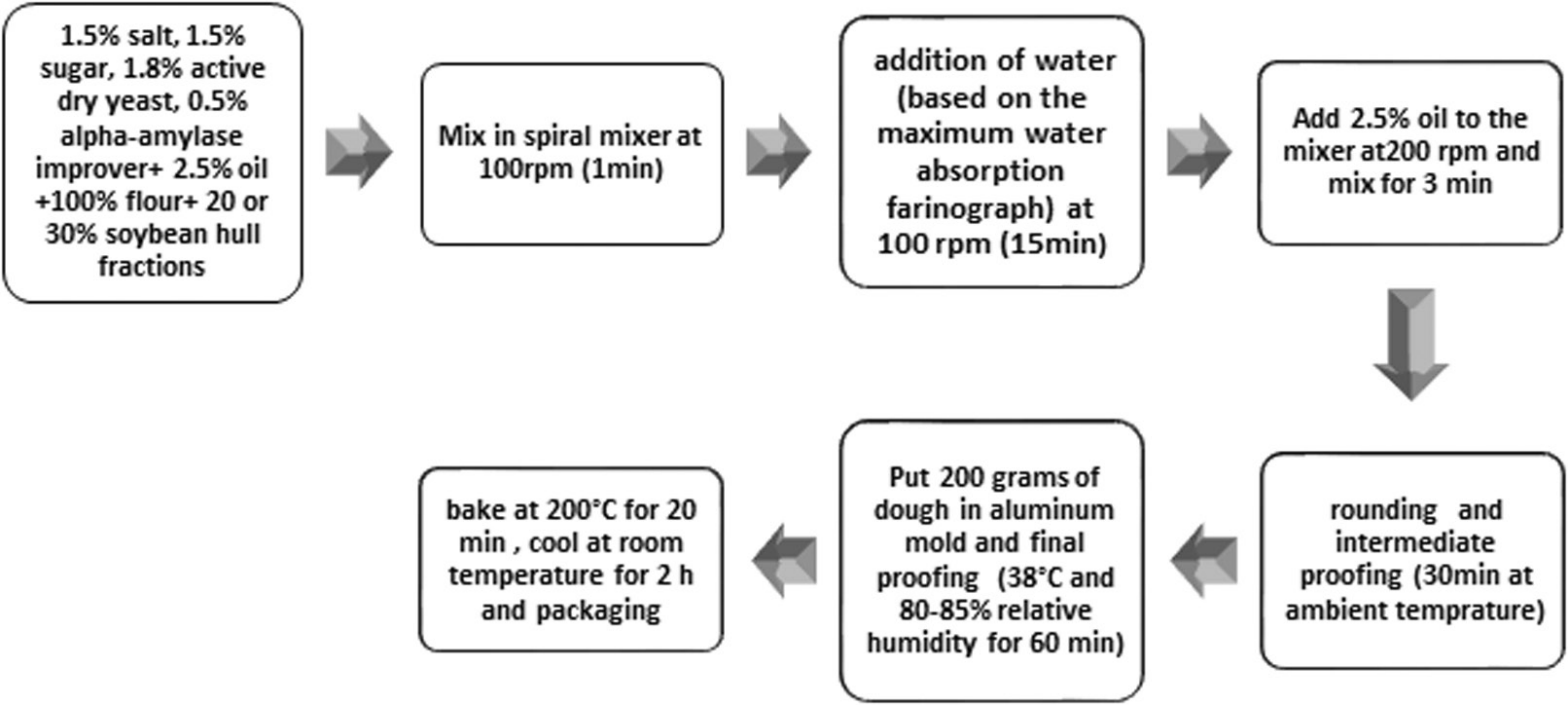
Diagram of preparation of dough and pan bread

All the dry ingredients were mixed with half of the oil in a spiral mixer (Hügel, No. HG550TMEM, Neuss, Germany) at 100 rpm for 1 min. The required water (based on the maximum water absorption of the samples in the farinograph curve) was gradually added to the mixture, and the dough was kneaded at 100 rpm for 15 min. The remaining oil was added to the dough, and then kneading was continued at 200 rpm for 3 min. The dough was taken out of the mixer, rounded and set aside at ambient temperature for 30 min (intermediate fermentation). Next, 200 g of the dough was placed in an aluminum pan (14 × 9 × 6 cm^3^) and fermented at 38°C and 80%–85% relative humidity for 60 min (EC160 incubator, Nuve company, Austin). The samples were then baked (LGH220, Irankhodsaz, Tehran, Iran) at 200°C for 20 min and cooled at room temperature for 2 h (Figure 1).

#### 2.2.3 Particle size analysis and chemical composition

The particle size distribution of the fractions of the soybean hull was analyzed in triplicate through laser light scattering using a Sald‐2101 particle sizer (Shimadzu, Japan). 0.5 g of the soybean hull was dispersed in 20 ml of distilled water. When the laser beam showed turbidity of 6%, the particle size measurement was performed in the sizes range of 0.01–1000 μm (Junejo et al., 2019).

The results were expressed as d (0.1), d (0.5), and d (0.9) corresponding to the maximum diameters of 10%, 50%, and 90% of the particles, respectively (in terms of percent total volume). Based on the particle size distribution, the specific surface area of the soybean hull fractions could be calculated as follows:

$$A_{p}\;\left(m^{-1}\right)=\frac{\text{Total area}}{\text{Total particle volume}}=\frac{\left[6\times \sum \left(\frac{V_{i}}{d_{i}}\right)\right]\;}{\sum v_{i.}},$$

where Ap is the specific surface area, *Vi* denotes the volume percentage of the particles, and di stands for the log average particle diameter in the sizes range resulting from light scattering (Noort et al., 2010).

Total dietary fiber (TDF), SDF, and IDF were quantified through the enzymatic–gravimetric method using a fiber assay kit (Megazyme K‐TDFR, Bray, Ireland; Tabibloghmany et al., 2020). All the chemical analyses were performed based on AACC (2000) standard methods.

#### 2.2.4 Farinographic characteristics

The dough mixing properties were determined using a Brabender farinograph (Farinograph‐E, 125, Germany) according to the method 54‐21.02 (AACC, 2000).

#### 2.2.5 Determination of chemical composition and specific volume of bread

Moisture, ash, and crude fiber contents were measured according to the 16A‐46, 1–8, 10–32 methods of AACC, respectively (AACC, 2000). The specific volume of the bread was quantified by the millet grain replacement method (10‐05‐1) of AACC (AACC, 2000). Moisture content was measured on the first, third, and fifth days after production.

#### 2.2.6 Texture profile analysis

Texture profile analysis (TPA) was done to examine the bread texture using a texture analyzer (Brookfield, model CTV1.5, the U.S.) with a 10‐kg load cell and a cylindrical flat‐ended probe (TA11/100 with a diameter of 35 mm and a length of 25.4 mm) on the first, third, and fifth days after production. The other parameters were as follows: probe speed of 1.00 mm/s, compression of 40%, and trigger force of 7 g. All the measurements were carried out in four replications using 2 × 2 × 2 cm^3^ blocks cut off the center of the bread loaves (Pauter et al., 2018; Steffe, 1996).

#### 2.2.7 Color assessment and image analysis of crumb structure

For color evaluation and image analysis of the bread, the images were captured using a digital camera (Canon EOS 1000D, Japan) at a lens aperture of 5.6, ISO of 100, and a shutter speed of 1/80 s to achieve high uniformity and reproducibility. Then, they were saved as the JPG format. The digital images were taken under five fluorescent lights (Opple, 8 W, model: MX396‐Y82; 60 cm in length). Image analysis was conducted using the ImageJ software (version 1.46r, National Institutes of Health, the U.S.) on a 30 × 50 mm^2^ area of the center of the bread loaf on the third day after production.

#### 2.2.8 Differential scanning calorimetry (DSC)

The bread treatments were cut off the center of the crumb. The amount of the sample was 15.8 ± 1.0 mg. This test was performed on the first, third, and fifth days after production. The scanning rate was 5°C min^−1^ from 25 to 160°C. The parameters of onset temperature, peak temperature, and enthalpy of melting were measured from the curve (Cai et al., 2014).

#### 2.2.9 X‐ray diffraction (XRD)

On the first, third, and fifth days after production, the samples were cut off the crumb center, dried (Drier oven, Parsazma, Tehran, Iran), and ground (Ball mill, Bosch, No. MKM6003, Germany). Then, the powdered samples were screened with a laboratory sieve (mesh No. of 60, the pore diameter of 250 μm) and subsequently introduced to XRD (X'Pert PRO MPD PANalytical Company, Nederland). The device was adjusted for a voltage of 40 kV and an intensity of 30 mA. Cu‐Ka irradiation was conducted at a wavelength of 01.54060 Ǻ. The diffractograms were obtained at 2Ө = 2–40 degrees and a speed of 2 degrees per min (Ribotta et al., 2004).

#### 2.2.10 Consumer‐oriented sensory evaluation

Sensory evaluation of the loaves of bread was done by the hedonic test and 20 panelists on the first, third, and fifth days after production. The sensory attributes were the shape and appearance, flavor, texture, and overall acceptability of the bread (the best = 5 and the worst = 1; Feili et al., 2013).

### 2.3 Statistical analysis

A completely randomized factorial design was created with the soybean hull type (extruded and non‐extruded), particle size (larger than 150 μm and smaller than 150 μm), and replacement level (20% and 30%) as the independent variables. All the measurements were triplicated and Duncan's multiple range test was used for mean comparison at a confidence level of 95%. A dough sample containing 20% wheat bran was also prepared to compare the behavior of the soybean hull with that of the wheat bran. The SPSS software version 23 was used to analyze the data.

## 3 RESULTS AND DISCUSSION

### 3.1 Particle size analysis and chemical composition

There was a positive correlation (*r* = .8) between the amounts of the ash and crude fiber of the different fractions of the soybean hull, as well as between the crude fiber and IDF (*r* = .962), which indicated that the crude fiber was generally representative of the IDF. The extruded soybean hull had a significantly lower ratio of IDF to SDF than the corresponding non‐extruded one (*p* < .05). The reduction in the particle size of both the extruded and non‐extruded samples increased the amount of SDF and decreased IDF content (Table S1).

The small‐sized fraction of the soybean hull had a larger specific surface area. The smallest particle size and the largest specific surface area were related to the extruded soybean hull under the sieve (ES). Moreover, the larger particles of the extruded soybean hull (EB) were significantly (*p* < .05) smaller than those of the non‐extruded one (BB) and had a larger surface area (Table 1).

**TABLE 1:** Chemical composition, particle size analysis, and specific surface area (m^−1^) of different samples (mean ± standard deviation)

	BB	BS	EB	ES	W
Parameters					
Humidity (%)	5.6 ± 0.07^a^	6.71 ± 0.1^b^	6.25 ± 0.05^d^	6.21 ± 0.03^e^	6.33 ± 0.1^c^
Protein (%)	12.08 ± 0.1^c^	16.38 ± 0.07^a^	11.97 ± 0.02^c^	16.37 ± 0.07^a^	13.56 ± 0.31^b^
Fat (%)	3.13 ± 0.05^e^	7.41 ± 0.03^a^	5.42 ± 0.17^c^	5.77 ± 0.06^b^	3.68 ± 0.03^d^
Ash (%)	5.33 ± 0.1^a^	4.01 ± 0.1^c^	4.03 ± 0.12^b^	4.52 ± ± 0.02^a^	3.68 ± 0.1^c^
Crude Fiber (%)	29.36 ± 0.22^a^	15.17 ± 0.219^c^	23.8 ± 1.3^b^	13.06 ± 0.1^d^	8.6 ± 0.52^e^
IDF (%)	64.76 ± 0.15^e^	55.86 ± 0.04^c^	57.99 ± 0.09^d^	53.03 ± 0.1^b^	47.6 ± 0.2^a^
SDF (%)	5.33 ± 0.25^e^	7.18 ± 0.08^d^	9.21 ± 0.09^b^	12.21 ± 0.1^a^	8.16 ± 0.2^c^
TDF (%)	70.10 ± 0.1^a^	63.04 ± 0.21^d^	67.20 ± 0.17^b^	65.24 ± 0.1^c^	55.76 ± 0.2^e^
IDF/SDF	12.16 ± 0.59^a^	7.77 ± 0.09^b^	6.29 ± 0.05^c^	4.33 ± 0.03^d^	5.79 ± 0.15^c^
Particle size analysis					
10% (μm)	241.441 ± 0.1^b^	58.563 ± 0.21^d^	217.316 ± 0.17^c^	16.145 ± 0.1^e^	295.192 ± 0.2^a^
50% (μm)	388.006 ± 0.25^b^	163.038 ± 0.08^d^	358.33 ± 0.09^c^	112.032 ± 0.1^e^	436.742 ± 0.2^a^
90% (μm)	560.446 ± 0.15^b^	275.49 ± 0.04^d^	513.39 ± 0.09^c^	225.358 ± 0.03^e^	587.79 ± 0.1^a^
AP (m^2^/m^3^)	229.59 ± 0.59^d^	257.77 ± 0.09^b^	234.155 ± 0.05^c^	410.118 ± 0.03^a^	225.032 ± 0.15^e^

*Note*: In each row, values with different lowercase letters have a significant difference (*p* < .05).

Abbreviations: BB, non‐extruded sample with large‐sized fractions; BS, non‐extruded sample with small‐sized fractions; EB, extruded sample with large‐sized fractions; ES, extruded sample with small‐sized fractions; W, control sample, wheat bran.

### 3.2 Farinographic characteristics

The size and amount of fiber had significant effects on water absorption and dough development time (*p* < 0.05; Table 2). In all our samples, the amount of water absorption significantly increased by increasing the amounts of the soybean hull fractions and decreasing the particle sizes. Generally, fiber absorbs more water than the main components of flour, namely, starch and protein. This effect can be due to a large number of hydroxyl groups in the structure of the soybean hull, which directly elevates water absorption by creating hydrogen bonds or retaining more water through capillary and adsorption properties (Noort et al., 2010). The maximum increase in water absorption was related to ES30 (Table S2). This sample had the smallest particle size and the highest SDF content. Furthermore, the extruded treatments had more amounts of SDF than the non‐extruded ones (Table S1). Based on previous results, under optimized extrusion conditions of the soybean hull, the amount of SDF is increased by the breakdown of the chemical bonds of IDF macromolecules. Furthermore, the dissociation of covalent and noncovalent bonds of protein and carbohydrate molecules with the fiber can lead to smaller and more soluble molecular fragments. Therefore, the extrusion process in the soybean hull increases the amount of soluble fiber and decreases the amount of insoluble fiber accordingly. By reducing the ratio of IDF/SDF, the water absorption index of the soybean hull increased (Tabibloghmany et al., 2020). The longer development time to reach the maximum consistency and the longer breakdown time by raising the amount of fiber could be owing to the fiber–gluten interactions. These interactions prevent the protein hydration process (Rosell et al., 2006). Moreover, some kinds of competition between the soybean hull and the other flour components for water absorption reduced the moisture content of the dough and thus strengthened its network. In all treatments excluding ES30, the stability time increased by rising in the amount of the soybean hull (*p* < .05) (Table S2). Although adding the soybean hull made the gluten network thinner, the interactions between the starch molecules and the soybean hull fiber could compensate for this deficiency. Raising the replacement level of the soybean hull caused decreasing in the dough's degree of softening. It can be associated with the rise in the non‐gluten protein content of the flour. These proteins may be involved in the development of the gluten network (Anjum et al., 2006). Compared with the other fractions of the soybean hull, BB samples, at both levels (20% and 30%) with the largest particle size, recorded the least stability time and the maximum degree of softening after 12 min (Table S2). The size elevating of the soybean hull particles probably prevented gluten network creation and subsequently the stability time shortening (Majzoobi et al., 2014).

**TABLE 2:** Effect of extrusion parameters, amount, and particle sizes of soybean hull fractions on Farinographic characteristics (mean ± standard deviation)

	Water absorption (%)	Dough development time (min)	Stability time (min)	Degree of softening (FU)	FQN Farinograph quality number (FU)	Break time (min)
BS30	70.6 ± 0.05^b^	5.60 ± 0.1^a^	6.00 ± 0.5^a^	42.00 ± 1.00^f^	80.66 ± 2.08^b^	7.98 ± 0.17^a^
BB30	68.76 ± 0.06^d^	5.10 ± 0.1^c^	4.13 ± 0.15^c^	65.66 ± 1.15^c^	69.33 ± 0.57^e^	6.06 ± 0.11^d^
BB20	65.31 ± 0.07^g^	3.4 ± 0.1^d^	3.96 ± 0.15^c^	76.33 ± 1.5^b^	63.00 ± 1.00^f^	6.30 ± 0.1^cd^
BS20	65.73 ± 0.3^b^	5.33 ± 0.05^b^	4.30 ± 0.26^c^	65.00 ± 3.00^c^	75.33 ± 0.57^cd^	7.30 ± 0.02^b^
ES30	71.70 ± 0.1^a^	5.70 ± 0.11^a^	4.23 ± 0.2^c^	52.33 ± 2.5^e^	81.00 ± 1.73^b^	7.57 ± 0.36^b^
ES20	68.85 ± 0.05^d^	5.26 ± 0.25^bc^	5.00 ± 0.11^b^	51.66 ± 3.07^e^	77.00 ± 1.05^c^	6.42 ± 0.14^c^
EB30	69.93 ± 0.1^c^	5.66 ± 0.06^a^	7.41 ± 0.12^a^	36.00 ± 3.6^g^	93.33 ± 2.8^a^	7.55 ± 0.045^b^
EB20	67.46 ± 0.25^e^	5.10 ± 0.1^c^	5.16 ± 0.06^b^	59.00 ± 1.10^d^	73.66 ± 1.15^d^	7.51 ± 0.02^b^
W20	65.15 ± 0.05^g^	2.66 ± 0.06^e^	3.90 ± 0.1^c^	82.66 ± 0.57^a^	57.00 ± 1.06^g^	6.28 ± 0.18^cd^

*Note*: In each column, values with different lowercase letters have a significant difference (*p* < .05). BB30 (non‐extruded sample with large‐sized fractions at 30%), BS20 (non‐extruded sample with small‐sized fractions at 20%), ES30 (extruded sample with small‐sized fractions at 30%), ES20 (extruded sample with small‐sized fractions at 20%), EB20 (extruded sample with large‐sized fractions at 20%), EB30 (extruded sample with large‐sized fractions at 30%), and W20 (control sample containing 20% wheat bran).

### 3.3 The moisture content of bread crumb

It was found out (Table 3) that the samples with smaller soybean hull particle sizes had a significantly higher moisture content than their corresponding ones with larger particle sizes (*p* < .05). Since finer particles had a larger surface area, higher SDF, and more hydrophilic groups (Table S1). According to the Farinograph test, these treatments absorbed more water in the dough preparation, and subsequently, this more water caused a rise in the moisture content of the final product (Tables 1 and 2; Marcin et al., 2016; Tabibloghmany et al., 2020). In all samples, by increasing the substitution level of the different fractions of the soybean hull, although the water absorption in the Farinograph test was significantly increased, the moisture content of the final product was reduced (*p* < 0.05) (Tables S2 and S3). In a way that there is a moderate and negative correlation between the amount of Farinograph water absorption and the moisture content of the bread crumb on the first, third, and fifth day after production (Table S3). This coefficient was −602, −670, and −617, respectively, on the first, third, and fifth day after production (*p* < .05). In this regard, researchers have reported a decrease in the moisture content of oat bran bread by increasing the replacement levels and particle sizes of the oat bran (Marcin et al., 2016). The highest rate of moisture loss was related to BS20 and ES20. Compared with the first day, the reduction in the moisture content on the fifth day was 8.23% and 7.82% for BS20 and ES20, respectively (Table S3). Although the smaller fiber particles, with a larger surface area, had a higher water‐binding capacity, water loss also was higher and faster than the other treatments during baking and storage. Therefore, their moisture loss during storage was more than the other bread (Marcin et al., 2016).

**TABLE 3:** Physicochemical properties of pan bread samples (all the values are in dry basis; mean ± standard deviation)

	Humidity% (First day)	Humidity% (Third day)	Humidity% (Fifth day)	Specific volume	Crude fiber (%)	Ash (%)
BS30	65.56 ± 1.00^Ac^	61.54 ± 2.08^Bc^	57.24 ± 0.17^Ce^	1.66 ± 0.5^e^	3.60 ± 0.1^b^	3.42 ± 0.05^a^
BB30	61.98 ± 0.11^Ad^	60.67 ± 0.57^Ac^	57.36 ± 0.11^Be^	1.663 ± 0.15^e^	5.7 ± 0.1^a^	2.90 ± 0.06^bc^
BB20	69.90 ± 0.10^Ab^	67.70 ± 1.00^Bb^	66.92 ± 0.1^Cb^	2.68 ± 0.15^bc^	3.48 ± 0.1^b^	2.21 ± 0.07^d^
BS20	70.20 ± 3.00^Ab^	68.12 ± 0.57^ABb^	67.22 ± 0.02^Bb^	2.64 ± 0.26^bc^	2.37 ± 0.05^d^	3.04 ± 0.3^bc^
ES30	65.79 ± 2.5^Ac^	62.06 ± 1.73^Bc^	61.51 ± 0.36^Ccd^	1.47 ± 0.20^e^	1.78 ± 0.11^e^	2.79 ± 0.1^c^
ES20	72.65 ± 3.07^Aa^	70.21 ± 1.05^Ba^	70.21 ± 0.14^Ca^	2.51 ± 0.11^c^	1.37 ± 0.25^f^	3.01 ± 0.05^bc^
EB30	64.77 ± 3.6^Ac^	61.44 ± 2.8^Bc^	58.53 ± 0.45^Cd^	1.95 ± 0.12^d^	3.65 ± 0.06^b^	3.13 ± 0.1^ab^
EB20	72.71 ± 1.1^Aa^	71.28 ± 1.15^Ba^	70.21 ± 0.02^Ba^	3.63 ± 0.06^a^	3.22 ± 0.1^c^	2.35 ± 0.25^d^
W20	71.95 ± 0.57^Aa^	67.92 ± 1.06^Bb^	66.50 ± 0.57^Ca^	2.84 ± 0.1^b^	1.26 ± 0.06^f^	3.25 ± 0.05^ab^

*Note*: Small shared letters indicate no significant differences in each column, and large shared letters indicate no significant difference in each row at 95% confidence level. BB30 (non‐extruded sample with large‐sized fractions at 30%), BS20 (non‐extruded sample with small‐sized fractions at 20%), ES30 (extruded sample with small‐sized fractions at 30%), ES20 (extruded sample with small‐sized fractions at 20%), EB20 (extruded sample with large‐sized fractions at 20%), EB30 (extruded sample with large‐sized fractions at 30%), and W20 (control sample containing 20% wheat bran).

### 3.4 Crude fiber, ash content, and specific volume of bread

According to the analysis of variance (Table 3), with an increase in the replacement level of the soybean hull and the particle sizes, the amounts of ash and crude fiber showed a significant increase in many cases, but the extruded treatments did not significantly change (*p* < .05). Compared with the other fractions, the highest amounts of crude fiber and ash were related to BB30, which contained the soybean hull fractions with the largest particle size and the highest content of IDF (Tables S1 and S3). In confirmation of these results, Majzoobi et al. (2013) declared that by reducing the particle sizes of wheat bran, the crude fiber and ash contents of the treatments decreased.

As the soybean hull particle sizes increased, the specific volume increased in all treatments, too. However, this increase was not significant in some cases (*p* < .05) (Table S3). The higher water absorption capacity of the smaller particles caused the rapid absorption of moisture by these components, and sufficient water did not remain for starch gelatinization, development of the gluten network, and absorption of the yeast nutrients. In addition, the smaller particles were more uniformly dispersed in the gluten network, resulting in more damage to the viscoelastic structure. This damage could lead to the network being unable to hold the released gases during fermentation (Noort et al., 2010; Tabibloghmany et al., 2020). Furthermore, by decreasing the particle sizes, IDF to SDF᾽s ratio was reduced. Researchers have shown that by raising the proportion of IDF to SDF, the specific volume of the final product increased, too (Bae & Lee, 2014). Elevation of the replacement level of the different fractions of the soybean hull reduced the volume of the final product due to both the dilution of the gluten network and the physical interactions or chemical reactions between the fiber, water, and gluten components (Anil, 2007). Mahasowanwong (1997) reported a decrease in the specific volume of molded bread with an increase in the level of soybean hull up to a maximum of 15% replacement (Mahasowanwong, 1997). In the present study, the effect of increasing the amount of soybean hull compared with the particle size was more significant. Besides, the increase in the substitution levels of dietary fiber decreased the amount of gluten. It also has affected the properties of the existing gluten. As a result, the gluten protein became firmer with less elasticity, and the gas storage capacity decreased (Kurek et al., 2016; Noort et al., 2010).

### 3.5 Color of bread crust and crumb

By reducing the particle sizes and applying the extrusion process, the darkness of the produced bread increased. The highest lightness (L*) was related to the non‐extruded soybean hull samples containing the large particle sizes, namely, BB30 and BB20. On the other hand, ES20 had the darkest crust (Table 4). It has been found out that the crust color alterations in the bread containing large amounts of fiber were influenced by the oxidation and releasing of the sugars that participate in caramelization during baking. In this regard, the changes that occurred during Maillard reactions were effective, as well (Marcin et al., 2016). Perhaps, one of the main reasons for the increase in the lightness of the high‐fiber samples was their significantly lower protein content (Feili et al., 2013). The smaller‐sized soybean hull fractions had a significant (*p* < .05) higher protein content than the larger sized ones that could be actively involved in the Maillard reactions (Table 1). In addition, the higher the loaf, the nearer to the upper heating radiation, and usually get darker color (Table S4). Extrusion affects the caramelization reactions by denaturing the proteins and releasing free sugars because of soybean hull cell wall disruption (Tabibloghmany et al., 2020; Yoo et al., 2011). Therefore, the released sugars during the extrusion of the soybean hull could react with amino acids. These released sugars cause the development of the Maillard reaction, less lightness, and greenness than the corresponding non‐extruded treatments. In the present study, with the elevation of the particle sizes, both a* and b* showed a significant decrease (*p* < .05) (Table S4). The rising occurrence of the browning reactions in the smaller particles probably caused the increase in redness and yellowness (Kurek et al., 2016).

**TABLE 4:** Effect of extrusion parameters, amount, and particle sizes of soybean hull fractions on L*, a*, and b* of crust and crumb in addition to crumb structural properties (mean ± standard deviation)

	BS30	BB30	BB20	BS20	ES30	ES20	EB30	EB20	W20
L* (crust)	59.63 ± 0.18^b^	69.75 ± 0.14^a^	60.37 ± 0.6^b^	52.30 ± 0.1^c^	49.65 ± 0.58^d^	49 ± 2.19^d^	58.80 ± 0.7^b^	50.84 ± 0.61^cd^	51.53 ± 1.91^c^
a* (crust)	2.99 ± 0.3^c^	−5.07 ± 0.12^i^	−2.05 ± 0.1^g^	1.14 ± 0.14^e^	4.15 ± 0.17^d^	3.57 ± 0.64^b^	−2.77 ± 0.1^h^	−0.986 ± 0.4^f^	2.39 ± 0.51^d^
b* (crust)	20.91 ± 0.1^c^	12.26 ± 0.27^h^	16.96 ± 0.23^f^	25.17 ± 0.49^b^	25.76 ± 0.19^a^	25.37 ± 0.4^ab^	14.76 ± 0.2^g^	18.35 ± 0.7^d^	17.57 ± 0.38^e^
L* (crumb)	79.13 ± 0.4^a^	76.94 ± 0.23^c^	75.15 ± 0.27^d^	76.58 ± 0.31^c^	74.48 ± 0.39^e^	74.99 ± 0.1^d^	70.82 ± 0.18^f^	74.13 ± 0.18^e^	77.45 ± 0.29^b^
a* (crumb)	−8.36 ± 0.8^d^	−8.47 ± 0.46^de^	−8.86 ± 0.11^e^	−8.6 ± 0.1^de^	−7.15 ± 0.2^b^	−7.56 ± 0.1^bc^	−7.60 ± 0.1^c^	−8.25 ± 0.57^d^	−6.62 ± 0.1^a^
b* (crumb)	3.15 ± 0.23^e^	1.86 ± 0.4^f^	1.41 ± 0.51^g^	2.03 ± 0.15^f^	6.72 ± 0.5^b^	6.10 ± 0.13^c^	5.96 ± 0.17^c^	5.30 ± 0.12^d^	6.48 ± 0.22^a^

	BS30	BB30	BB20	BS20	ES30	ES20	EB30	EB20	W20
Count	897 ± 3.51^b^	863.30 ± 2.51^c^	597.0 ± 1^f^	702 ± 7^e^	1194.3 ± 1.53^a^	754 ± 4^d^	862 ± 2^c^	506.34 ± 8.5^g^	426.33 ± 1.53^h^
Total area (mm)^2^	126.75 ± 2.21^f^	120.71 ± 2.66^g^	185.90 ± 0.39^c^	158.80 ± 1.71^d^	111.14 ± 1.35^h^	154.04 ± 4.77^e^	128.36 ± 0.72^f^	192.47 ± 2.95^b^	209.23 ± 2.02^a^
Average pore size (mm)	0.105 ± 0.002^h^	0.129 ± 0.002^g^	0.236 ± 0.005^d^	0.216 ± 0.001^f^	0.172 ± 0.002^d^	0.215 ± 0.002^e^	0.203 ± 0.007^a^	0.38 ± 0.001^b^	0.372 ± 0.002^b^
Porosity%	25.83 ± 0.31^f^	26 ± 0.8^f^	35.65 ± 0.62^c^	31.47 ± 0.61^d^	20.43 ± 0.37^g^	30.12 ± 0.6^e^	29.42 ± 0.22^e^	44.73 ± 0.7^a^	37.37 ± 0.4^b^
P < 2 mm	885.66 ± 3.05^b^	851.67 ± 1.52^c^	579.34 ± 1.15^f^	684.67 ± 7.09^e^	1185.33 ± 1.73^a^	740.67 ± 4.04^d^	851 ± 2.0^c^	481.33 ± 9.53^g^	411.66 ± 1.73^h^
P < 10 mm < 2 mm	11 ± 1^d^	11.34 ± 0.6^d^	17.67 ± 0.6^b^	17.33 ± 1.15^c^	9.0 ± 0.1^e^	13.33 ± 0.57^c^	10.67 ± 0.6^d^	.24.66 ± 0.58^a^	14.00 ± 1^c^

*Note*: Small shared letters indicate no significant differences in each row at 95% confidence level. BB30 (non‐extruded sample with large‐sized fractions at 30%), BS20 (non‐extruded sample with small‐sized fractions at 20%), ES30 (extruded sample with small‐sized fractions at 30%), ES20 (extruded sample with small‐sized fractions at 20%), EB20 (extruded sample with large‐sized fractions at 20%), EB30 (extruded sample with large‐sized fractions at 30%), and W20 (control sample containing 20% wheat bran).

The color changes in bread are only affected by the browning and caramelization reactions in the crust. In the crumb of the bread, since the required temperatures of these reactions are not provided, the color changes are correlated with the dough ingredients (Marcin et al., 2016).

The highest and the lowest bread crumb lightness pertained to BS30 and EB30, respectively (Table 4). Moreover, these two fractions ‐BS, EB‐ were also the lightest and the darkest components of the soybean hull other than the bread formulation. Almeida et al. (2013) also indicated that the color changes in the crumb of bread were because of the nature and inherent color of the dietary fiber components used in the bread formula. Under the influence of the soybean hull extrusion, its lightness decreased, whereas its yellowness and redness increased (Kurek et al., 2016; Tabibloghmany et al., 2020), as these color changes were observed in all the extruded samples compared with the corresponding non‐extruded ones.

### 3.6 Structure of bread crumb

With a rise in the amount of the replaced soybean hull and a reduction in the particle sizes, the cells number in the crumb of the bread significantly increased (*p* < .05; Table 4, Figure S2). So the maximum cells number was related to ES30 with the minimum particle sizes and the highest level of soybean hull replacement. A large number of the cells per unit area creates a small average cell size, resulting in low porosity and a compact texture. Thus, the crumb treatments with the minimum number of cells per unit area showed the maximum mean cell size and porosity percentage (Table S4; Figure S2). Amir et al. (2013) also reported similar results.

Bread crumb image processing showed that reducing the particle sizes of the soybean hull, and more importantly, increasing the amount of soybean hull decreased the ability of the dough to retain gas (Figure S2). Thus, it created a denser bread with a smaller specific volume. In all the extruded samples, compared with the non‐extruded ones, the percentage of porosity decreased or did not significantly change except for EB20. This latter treatment showed the minimum cell numbers, the maximum total area, and the highest percentage of porosity. The reduction in the porosity percentage was significant, especially in the extruded treatments with finer particle sizes compared with the corresponding non‐extruded ones (*p* < .05) (Table S4; Figure S2).

Polaki et al. (2010) demonstrated that the cells in the crumb structure bread with diameters less than 4 mm, and more than 8 mm were as small and large, respectively.

**FIGURE 2:**
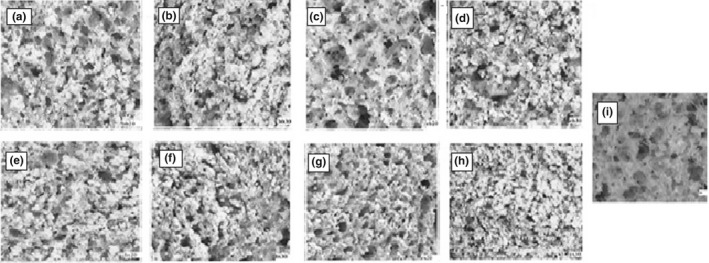
Eight‐bit images of bread crumbs: (a) BB20, (b) BB30, (c) EB20, (d) EB30, (e) BS20, (f) BS30, (g) ES20, (h) ES30, and (i) W20, BB30 (non‐extruded sample with large‐sized fractions at 30%), BS20 (non‐extruded sample with small‐sized fractions at 20%), ES30 (extruded sample with small‐sized fractions at 30%), ES20 (extruded sample with small‐sized fractions at 20%), EB20 (extruded sample with large‐sized fractions at 20%), EB30 (extruded sample with large‐sized fractions at 30%), and W20 (control sample containing 20% wheat bran)

Based on the analysis of variance results (Table 4), the cell size distribution in all the bread treatments was small cells. And more than 90% of the cells in the crumb structure bread had diameters less than 2 mm. The maximum number of the small cells was in ES30 with a compact internal texture. Furthermore, the maximum number of the cells with diameters below 10 mm was related to EB20 with the most porous crumb (Figure 2). Moreover, there was a strong Pearson correlation (*r* = .936) between the specific volume of the bread and the porosity percentage (Table S4). In this regard, Kurek and Wyrwisz (2015) reported similar results.

### 3.7 TPA

**FIGURE 3:**
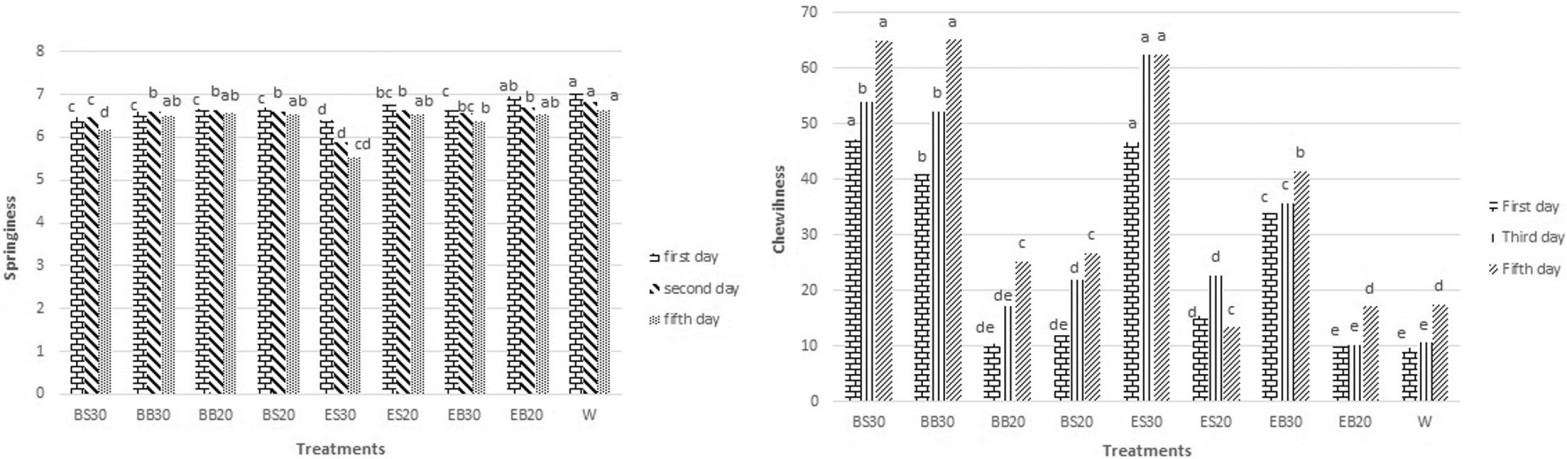
Effect of extrusion parameters, amount, and particle sizes of soybean hull fractions on springiness, and chewiness during storage, BB30 (non‐extruded sample with large‐sized fractions at 30%), BS20 (non‐extruded sample with small‐sized fractions at 20%), ES30 (extruded sample with small‐sized fractions at 30%), ES20 (extruded sample with small‐sized fractions at 20%), EB20 (extruded sample with large‐sized fractions at 20%), EB30 (extruded sample with large‐sized fractions at 30%), and W20 (control sample containing 20% wheat bran)

The crumb hardness and elasticity of all treatments significantly increased by increasing the replacement level of the soybean hull (*p* < 0.05; Table 5; Figure 3). The variations in chewiness are influenced by changes in hardness, cohesiveness, and springiness. The bread with the highest hardness had the maximum chewiness (Figure 3). By elevating the replacement level of the soybean hull and decreasing its particle sizes, the chewiness increased. This parameter increased over time. The increased hardness by raising the amount of the fiber could be due to the limited expansion of gas bubbles because of the presence of the different fractions of the soybean hull (Amir et al., 2013; Tuncel et al., 2014). In many cases, the crumb hardness decreased over time, and its cohesiveness rose with an increase in the particle sizes. In all conditions, the highest values of cohesiveness and elasticity belonged to EB20 (Table 5; Figure 3). The decrease in the firmness of the crumb of bread by increasing the particle sizes of β‐glucan in wheat bread roll is consistent with the results of the present study (Marcin et al., 2016; Skendi et al., 2010). Furthermore, in the bread containing the small‐sized soybean hull fractions, treatments with the extruded soybean hull fractions had more hardness than the non‐extruded ones during the storage (Table S5). Thus, ES30 had the highest crumb hardness among all treatments in three periods. Gómez et al. (2011) also reported an increase in the hardness of the bread crumb containing extruded wheat bran compared with one containing the non‐extruded bran (Gómez et al., 2011). There was a significant raise in the farinograph quality number (FQN; Table 3), with an increase in the amount of the soybean hull fractions (*p* < .05), especially those with small particle sizes. However, the produced dough had excessive stiffness. The high stiffness of the dough increased the wall thickness surrounding the air cells, decreased the air bubbles rising, and consequently elevated the crumb hardness (Table S5). In this regard, other researchers reported by increasing the replacement level and reducing the particle sizes of wheat bran in pan bread, the bread was produced with a much denser structure, less specific volume, and ultimately more firmness (Sehn & Steel, 2020). In the present study, EB20 had the lowest crumb hardness with the highest specific volume at all three periods (Tables S3 and S5). There was a negative correlation between the specific volume of the bread containing dietary fiber and its hardness (Gómez et al., 2003; Tuncel et al., 2014). Moreover, in our study, there was a strong and negative correlation between the specific volume and hardness of the bread crumb during storage. In this regard, the correlation coefficients were equal to −0.904, −0.892, and −0.876 on the first, third, and fifth days, respectively. Also, there is a strong and negative correlation between sensory acceptance of texture properties and the degree of hardness of bread texture. In this way, the higher hardness of the bread texture, the lower the sensory score of the bread texture. The correlation coefficient between hardness measured with the texture analyzer and its sensory score was on the first, third, and fifth days (−0.745), (−0.832), and (−0.812), respectively.

**TABLE 5:** Effect of extrusion parameters, amount and particle sizes of soybean hull fractions on hardness and cohesiveness during storage (mean ± standard deviation)

	Hardness (g)			Cohesiveness		
Samples	First day	Third day	Fifth day	First day	Third day	Fifth day
BS30	1378.33 ± 3.51^Cb^	2166.34 ± 236.5^Bb^	3275.00 ± 365^Aa^	0.516 ± 0.15^Aabc^	0.383 ± 0.02^Bcd^	0.325 ± 0.03^Cc^
BB30	1265.34 ± 212.07^Cd^	1955.00 ± 58^Bb^	2704.50 ± 105.5^Ab^	0.49 ± 0.03^Abcd^	0.405 ± 0.005^Bbc^	0.37 ± 0.01^Babc^
BB20	299.67 ± 8.73^Cde^	560.00 ± 10^Bd^	970.00 ± 100^Ae^	0.535 ± 0.015^Aa^	0.460 ± 0.01^Bb^	0.40 ± 0.01^Cab^
BS20	330.00 ± 20^Cde^	699.33 ± 16.77^Bd^	1018.14 ± 128.10^Ad^	0.537 ± 0.015^Aa^	0.48 ± 0.04^Bb^	0.395 ± 0.015^Cab^
ES30	1555.00 ± 25^Ca^	2934.00 ± 267^Ba^	3300.00 ± 160^Aa^	0.47 ± 0.03^Ad^	0.36 ± 0.02^Bd^	0.356 ± 0.025^Bbc^
ES20	432.00 ± 75.74^Cd^	721.00 ± 101^Bd^	1051.62 ± 122.77^Ad^	0.526 ± 0.02^Aab^	0.473 ± 0.005^Ba^	0.385 ± 0.005^Cab^
EB30	1056.67 ± 101.15^Bc^	1246.50 ± 58.51^Bc^	1710.00 ± 160^Ac^	0.483 ± 0.011^Abcd^	0.423 ± 0.006^Bb^	0.38 ± 0.02^Cab^
EB20	258.00 ± 18.35^Ce^	324.34 ± 21.82^Be^	622.02 ± 2.03^Ae^	0.547 ± 0.011^Aa^	0.486 ± 0.015^Ba^	0.42 ± 0.06^Ba^
W20	262.34 ± 19.13^Ce^	329.00 ± 21.51^Be^	670.58 ± 10.19^Ae^	0.536 ± 0.015^Aa^	0.480 ± 0.01^Ba^	0.39 ± 0.01^Cab^

*Note*: Small shared letters indicate no significant differences in each column, and large shared letters indicate no significant difference in each row at 95% confidence level. BB30 (non‐extruded sample with large‐sized fractions at 30%), BS20 (non‐extruded sample with small‐sized fractions at 20%), ES30 (extruded sample with small‐sized fractions at 30%), ES20 (extruded sample with small‐sized fractions at 20%), EB20 (extruded sample with large‐sized fractions at 20%), EB30 (extruded sample with large‐sized fractions at 30%), and W20 (control sample containing 20% wheat bran).

### 3.8 DSC

**FIGURE 4:**
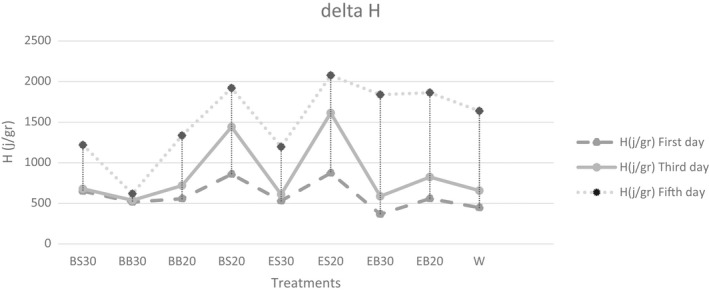
Effect of extrusion parameters, amount, and particle sizes of soybean hull fractions on enthalpy during storage, BB30 (non‐extruded sample with large‐sized fractions at 30%), BS20 (non‐extruded sample with small‐sized fractions at 20%), ES30 (extruded sample with small‐sized fractions at 30%), ES20 (extruded sample with small‐sized fractions at 20%), EB20 (extruded sample with large‐sized fractions at 20%), EB30 (extruded sample with large‐sized fractions at 30%), and W20 (control sample containing 20% wheat bran)

In all treatments, with a rise in the soybean hull replacement level and a decrease in the particle sizes of the different fractions of the soybean hull, the onset temperature increased (Table 6). In addition, this temperature increased in the treatments with the extruded fractions soybean hull compared with the ones including the non‐extruded fractions (Table S6). Enthalpy and peak temperature increased as the particle sizes decreased in the different fractions of the soybean hull; however, these two parameters decreased in many cases by raising the replacement level. All the parameters of the onset temperature, peak temperature, and enthalpy increased over time from the first to the fifth day (Table S6; Figure 4). In the present study, BB30 had the largest particle sizes, the maximum amount of IDF, and the lowest enthalpy at all three periods (Tables S1 and S6). In the latter treatment, probably higher levels of IDF prevented amylopectin retrogradation. IDF inhibits any bond formation between the amylose and amylopectin molecules. Moreover, the lower enthalpy values may be due to the dilution effect of starch on these samples (Sehn & Steel, 2020). The dilution of the gelatinized starch reduced the availability of starch for crystallization. The thinner starch, the fewer crystals formed; as a result, the peak temperature will drop. Inconsistent with these results, other researchers indicated that compared with the other dietary fiber mixtures, the addition of the various types of dietary fiber with large particle sizes containing more IDF to wheat bread formulation significantly reduced the enthalpy and slowed down the starch retrogradation (Cai et al., 2014; Santos et al., 2008). In our research, it seems that raising the SDF content and decreasing the particle sizes of the treatments containing the extruded soybean hull were the reasons for the higher peak temperature of the crystals in these treatments.

**TABLE 6:** Effect of extrusion parameters, amount, and particle sizes of soybean hull fractions on differential scanning calorimetry parameters during storage (mean ± standard deviation)

Samples	To (First day)	To (Third day)	To (Fifth day)	Tp (First day)	Tp (Third day)	Tp (Fifth day)	H (j/gr) First day	H (j/gr) Third day	H (j/gr) Fifth day
BS30	40.10 ± 0.7^Bb^	42.73 ± 0.15^Ab^	43.16 ± 0.06^Ab^	73.40 ± 0.87^Cb^	75.32 ± 0.22^Bb^	80.1 ± 0.19^Ab^	650.55 ± 1.86^Cc^	679.15 ± 3.37^Be^	1220.15 ± 6.51^Ae^
BB30	33.07 ± 0.08^Ce^	40.20 ± 0.3^Bd^	42.00 ± 0.8^Acd^	67.85 ± 0.55^Cd^	69.57 ± 0.21^Bf^	65.06 ± 0.3^Aa^	516.31 ± 0.9^Cf^	537.80 ± 7.9^Bi^	618.72 ± 9.78^Af^
BB20	38.93 ± 0.17^Cbc^	34.93 ± 0.35^Bf^	38.71 ± 0.39^Ag^	69.60 ± 0.50^Cc^	71.60 ± 1.4^Be^	75.65 ± 0.44^Acd^	558.22 ± 2.01^Cd^	719.30 ± 1.96^Bd^	1336.31 ± 13.5^Ae^
BS20	37.84 ± 2.76^Bc^	40.85 ± 0.99^Abcd^	41.51 ± 0.28^Acd^	77.44 ± 0.4^Cb^	76.00 ± 0.1^Bb^	82.30 ± 0.1^Aa^	859.74 ± 2.60^Cb^	1442. ±17.75^Bb^	1921.10 ± 13.15^Ab^
ES30	42.15 ± 0.06^Ca^	45.96 ± 0.58^Ba^	50.83 ± 0.56^Aa^	73.36 ± 0.72^Bb^	74.40 ± 0.52^Bcd^	76.3 ± 0.4^Ac^	532.42 ± 1.03^Ce^	610.57 ± 1.87^Bg^	1197.15 ± 3.03^Ae^
ES20	38.90 ± 0.3^Cc^	41.61 ± 0.27^Bc^	42.11 ± 0.1^Acd^	75.33 ± 0.96^Ba^	77.34 ± 1.05^Ba^	82.33 ± 0.45^Aa^	874.22 ± 0.36^Ca^	1613.8 ± 14.10^Ba^	2076.83 ± 83.54^Aa^
EB30	39.48 ± 0.3^Cbc^	40.42 ± 0.19^Bd^	42.71 ± 0.59^Abc^	69.54 ± 0.16^Ac^	70.85 ± 0.63^Ce^	75.00 ± 0.1^Bd^	367.53 ± 0.41^Ch^	586.81 ± 4.82^Bh^	1839.67 ± 4.42^Ac^
EB20	36.03 ± 0.6^Cd^	38.81 ± 0.24^Be^	40.64 ± 0.11^Ae^	70.48 ± 1.01^Bc^	73.58 ± 0.62^Ce^	78.93 ± 1.15^Bb.^	558.95 ± 0.09^Cd^	824.6 ± 0.48^Bc^	1863.63 ± 18.95^Ac^
W20	34.82 ± 0.7^Cd^	38.22 ± 0.26^Be^	39.81 ± 0.55^Af^	72.37 ± 0.2^Bb^	75.93 ± 0.65^Cc^	79.13 ± 1.4^Bb^	448.33 ± 0.21^Cg^	656.9 ± 22.08^Bf^	1638.59 ± 4.28^Ad^

*Note*: Small shared letters indicate no significant differences in each column, and large shared letters indicate no significant difference in each row at 95% confidence level. BB30 (non‐extruded sample with large‐sized fractions at 30%), BS20 (non‐extruded sample with small‐sized fractions at 20%), ES30 (extruded sample with small‐sized fractions at 30%), ES20 (extruded sample with small‐sized fractions at 20%), EB20 (extruded sample with large‐sized fractions at 20%), EB30 (extruded sample with large‐sized fractions at 30%), and W20 (control sample containing 20% wheat bran).

Abbreviations: H, enthalpy; To, onset temperature; Tp, peak temperature.

Recrystallization increases as the maximum moisture content elevated to 45%–50%, which boosts the lubricating effect and mobility of the molecules (Santos et al., 2008). There is a strong positive and significant correlation between the moisture content of treatments and enthalpy changes in this study, especially on the third and fifth days after production (Table S3; Figure S4). The correlation coefficient between enthalpy changes and moisture content on the third and fifth days are (*r* = .618) and (*r* = .694) respectively (Santos et al., 2008).

### 3.9 XRD

Based on the obtained diffractograms, the maximum signal intensities of the samples with 20% soybean hull and wheat bran were at the Bragg angle in the range of 19.77° to 20.06° (Table S7; Figure S5). Nonetheless, in the treatments with 30% of the different fractions of the soybean hull, the highest peak was in the angle range of 26.60°–29.28° on the first day after production (Table 7).

**TABLE 7:** Effect of extrusion parameters, amount, and particle sizes of soybean hull fractions on maximum signal intensity (count/s) of XRD patterns during storage (mean ± standard deviation)

Treatment	First day	Third day	Fifth day
BS30	26.06 ± 0.66^Af^	30.20 ± 0.25^Bf^	38.39 ± 0.75^Cf^
BB30	28.15 ± 0.51^Ae^	30.47 ± 0.28^Bf^	35.93 ± 0.39^Cf^
BB20	39.00 ± 0.1^Abc^	43.49 ± 1.2^Bd^	77.47 ± 1.25^Cc^
BS20	39.38 ± 0.16^Ab^	55.76 ± 2.99^Bb^	83.30 ± 3.02^Cb^
ES30	24.80 ± 0.17^Ag^	38.75 ± 1.12^Be^	42.95 ± 2.94^Ce^
ES20	40.04 ± 0.077^Aa^	61.88 ± 0.06^Ba^	98.18 ± 0.12^Ca^
EB30	25.86 ± 0.34^Af^	30.97 ± 0.13^Bf^	37.72 ± 1.32^Cf^
EB20	38.66 ± 0.41^Ac^	42.83 ± 0.68^Bd^	70.59 ± 0.72^Cd^
W20	37.61 ± 0.39^Ad^	51.34 ± 0.96^Bc^	84.18 ± 0.72^Cb^

*Note*: Small shared letters indicate no significant differences in each column at 95% confidence level. BB30 (non‐extruded sample with large‐sized fractions at 30%), BS20 (non‐extruded sample with small‐sized fractions at 20%), ES30 (extruded sample with small‐sized fractions at 30%), ES20 (extruded sample with small‐sized fractions at 20%), EB20 (extruded sample with large‐sized fractions at 20%), EB30 (extruded sample with large‐sized fractions at 30%), and W20 (control sample containing 20% wheat bran).

Analysis of the diffraction patterns revealed that the distance between the crystal plates (d‐spacing) was approximately 4.4 Å, equivalent to the Bragg angle of 20°, which determines the crystal type V. This crystal is a result of the helical clathrates of amylose complex with fatty acids (Ribotta et al., 2004).

Although this angle range was also present in the diffractograms of the treatments containing 30% soybean hull, the maximum signal intensity did not lie in it. It seems that the highest peak, recorded in the range of 26°–29° on the first day, was related to the cellulose crystals in the IDF of the different fractions of the soybean hull. In agreement with this result, Lamsal et al. (2010) reported the maximum signal intensity for different soybean hull fractions at 26° (Lamsal et al., 2010). The position of the peak shifted to the range angles of 19.7°–20° on the third day after production in the treatments with 30% hull. However, in the range of 15°–17°, the peaks were also recorded. At the same time, the signal intensity was not maximum at these angles. On the fifth day after production, in many cases, the position of the highest peak shifted to 16.96°–17.12°, but in some of them, such as EB30 and ES30, it was still at 20° (Table S7). The peak formation at the angle of 15° or 17° during bread storage indicates the formation of β‐type crystal structure (Ribotta et al., 2004). This structure represents the retrogradation of amylopectin during storage.

The maximum signal intensity on the third and fifth days after production increased with reducing the particle sizes of the different fractions of the soybean hull; yet, it decreased with raising the replacement level in many cases (Table S7). Compared with the treatments with the lower amount of the soybean hull fractions, the corresponding ones with the higher amounts had less amylose, amylopectin, starch available for crystallization. Consequently, the maximum intensity was reduced in their diffractograms, as well. These findings were consistent with the results accomplished by Yildiz et al. (2016).

Furthermore, by increasing the particle sizes of dietary fibers, complicated complexes may be formed between the fiber particles and amylose. These complex prevent the accumulation of amylose and amylopectin particles and recrystallization (Demirkesen et al., 2014; Ribotta et al., 2004; Yildiz et al., 2016). The highest peaks of the treatments with 30% of the different fractions of the soybean hull probably pertain to the cellulose crystals.

By reducing the particle sizes and applying the extrusion process, the cellulose crystalline fractions were reduced in the dietary fiber of the soybean hull, which is probably the reason for the significant reduction in the maximum signal intensity in EB30 and ES30 on the fifth day, compared with BB30 and BS30 (*p* < .05) (Table S7).

**FIGURE 5:**
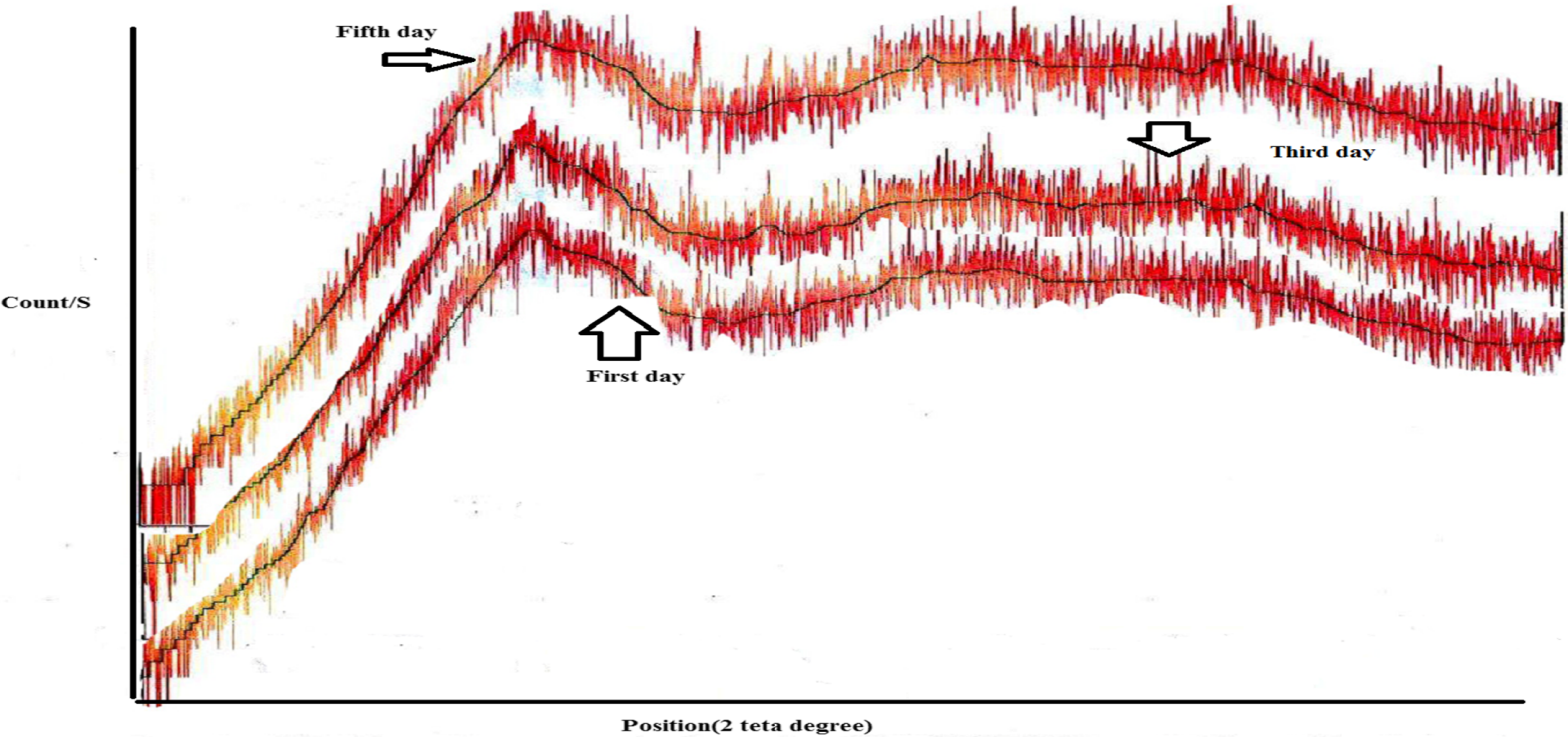
Changes in XRD pattern of BS20 during storage, BS20 (non‐extruded sample with small‐sized fractions at 20%)

The increased crystallization of starch during storage caused a significant increase in the signal intensity (count per second) in all the samples (Figure 5). Many researchers have reported an increase in the maximum signal intensity during the storage (Demirkesen et al., 2014; Ribotta et al., 2004; Yildiz et al., 2016).

X‐ray crystallography may be correlated with DSC for measuring the increase in crystallinity during storage (Ghoshal et al., 2016). In this study, there was a moderate positive correlation between the enthalpy and the maximum signal intensity at three periods. The correlation coefficients between these two variables on the first, third, and fifth days after production were 0.5, 0.834, and 0.660, respectively (Tables S6 and S7).

### 3.10 Consumer‐oriented sensory evaluation

Sensory properties are greatly affected by the presence of dietary fiber in the structure of bread. Many studies have shown that the presence of high levels of dietary fiber, regardless of the source, leads to the overall rejection of the product by the consumers. The acceptable amount of dietary fiber incorporated in the formula in many studies has been between 5% and 15% (Ktenioudaki & Gallagher, 2012).

**FIGURE 6:**
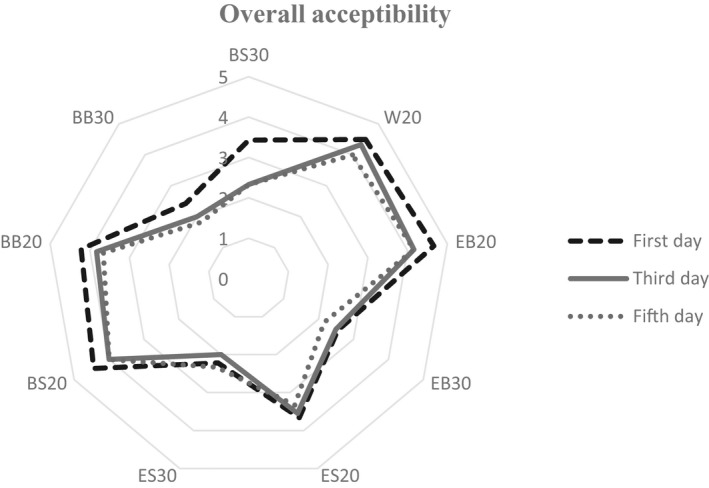
Effect of extrusion parameters, amount, and particle sizes of soybean hull fractions on sensory attributes and overall acceptance during storage, BB30 (non‐extruded sample with large‐sized fractions at 30%), BS20 (non‐extruded sample with small‐sized fractions at 20%), ES30 (extruded sample with small‐sized fractions at 30%), ES20 (extruded sample with small‐sized fractions at 20%), EB20 (extruded sample with large‐sized fractions at 20%), EB30 (extruded sample with large‐sized fractions at 30%), and W20 (control sample containing 20% wheat bran)

However, in the present study, based on the results of the analysis of variance of the sensory scores at all three periods, all treatments containing 20% soybean hull with different particle sizes as well as W20 acquired acceptable scores in terms of overall acceptance. At all three periods, all the sensory scores significantly decreased (*p* < 0.05) by increasing the level of soybean hull replacement from 20% to 30% (Figure 6).

Decreased acceptance of sensory characteristics of the bread can be attributed to the firmness of the treatments containing more soybean hull because of the decrease in the gluten content of the bread. In the treatments comprising higher dietary fiber levels, the inadequate association in the gluten network and the dilution of starch in the presence of the fiber components reduced the amount of the available starch for gelatinization, the elasticity of the dough, and the flexibility of the bread, resulting in the early staling, more hardness in texture and increased chewiness of the bread (Sehn & Steel, 2020). There is a strong and negative Pearson's correlation between general acceptance characteristics and hardness of texture (TPA) in all periods. The correlations between overall acceptance and hardness on the first, third, and fifth days after production are (*r* = −.780), (*r* = −.811) and (*r* = −.755), respectively.

Although W20 and EB20 were the best in all the sensory attributes during evaluations, no significant difference was observed between W20 and the treatments containing 20% of the soybean hull regardless of the particle sizes and extrusion (*p* < .05) (Figure S6).

Although the sensory scores decreased over time, this reduction was not significant in many cases (*p* < .05). The overall acceptance of EB20 and BS20 did not change, and both of them gained the same score even on the fifth day, compared with the third day after production. This result showed the acceptable shelf‐life of the produced bread and its freshness owing to the presence of the dietary fiber particles in the formulation.

## 4 CONCLUSIONS

Different fractions of extruded and non‐extruded soybean hull with different particle sizes at 20% and 30% of the bread formula affected its physicochemical and sensory properties.

Adding more than 15% of the different fractions of the extruded soybean hull, especially the large‐sized fractions, to wheat bread increases the bread health properties and promotes textural, sensory, and storage characteristics. Although the extrusion process of soybean hull and separation of the large‐sized fractions may not be economical, the growing tendency to consume healthy and low‐calorie foods shows a clear prospect for producing such products.

Investigation of the use of emulsifiers, especially sodium stearoyl lactylate in the bread formulated with extruded fractions of soybean hull and their effects on the physicochemical and sensory properties of the bread can be suggested as a useful work for further research.

## CONFLICT OF INTEREST

The authors declare that they have no conflict of interest.

## ETHICS STATEMENT

This article does not contain any studies with human or animal subjects.

## Supporting information


Appendix S1

## Data Availability

The data that support the findings of this study are openly available in the central library at Ferdowsi University. The authors confirm that the data supporting the findings of this study are available within the article and its supporting information.
