# Supplementary material for: Effects of substitution level and particle size of extruded soybean hull fractions on physicochemical and sensorial properties of high‐fiber pan bread during storage
Source: Food Sci Nutr. 2022 Oct 6;10(12):4345–59. doi: 10.1002/fsn3.3027 (PMC9731523; doi:10.1002/fsn3.3027)
Supplement: Supplementary file 1 — Appendix S1 [file FSN3-10-4345-s001.docx]

Table 1 Effect of extrusion parameters, amount and particle size of the soybean hull fractions on gumminess and chewiness during storage (mean ± standard deviation)

|  | **Gumminess (gram)** | | | **Chewiness (** **millijoules )** | | |
| --- | --- | --- | --- | --- | --- | --- |
| **Samples** | **First day** | **Third day** | **fifth day** | **First day** | **Third day** | **Fifth day** |
| **BS30** | **712 ±21.65^Ba*^** | **832.52 ± 125.07 ^Bb^** | **1055.67 ± 8.02 ^Ab^** | **47.27 ± 1.33 ^Ba^** | **53.84 ± 7.78 ^Bb^** | **65.02 ± 0.54 ^Aa^** |
| **BB30** | **617.33 ± 82.09 ^Cb^** | **791.67 ± 13.5 ^Bb^** | **999.33 ± 12.01^Ab^** | **40.81 ± 5.00 ^Cb^** | **52.196 ± 0.93 ^Bb^** | **65.06 ± 1.53 ^Aa^** |
| **BB20** | **156.50 ± 8.26 ^Cde^** | **257.66 ± 8.96 ^Bde^** | **387.33 ± 30.00 ^Ad^** | **10.43 ± 0.64 ^Cde^** | **17.10 ± 0.41^Bde^** | **25.25 ± 1.83 ^Ac^** |
| **BS20** | **177.33 ± 10.11 ^Cde^** | **335.16 ± 21.65 ^Bd^** | **409.67 ± 58.58 ^Ad^** | **11.88 ± 0.91 ^Cde^** | **21.98 ± 1.27 ^Bd^** | **26.75 ± 3.64 ^Ac^** |
| **ES30** | **730.00 ± 44.44 ^Ba^** | **1059.67 ± 154.53 ^Aa^** | **1231 ± 33.15 ^Aa^** | **46.72 ± 4.72 ^Ba^** | **62.39 ± 9.22 ^Aa^** | **62.44 ± 1.84 ^Aa^** |
| **ES20** | **226.66 ± 31.87 ^Bd^** | **341.66 ± 52.05 ^Ad^** | **405 ± 52.56 ^Ad^** | **15.34 ± 2.01 ^Bd^** | **22.67 ± 3.25 ^Ad^** | **13.33 ± 2.95 ^Ac^** |
| **EB30** | **511.67 ±60.38 ^Bc^** | **542.00 ± 23.06 ^ABc^** | **652 ± 95.01 ^Ac^** | **34.00 ± 4.03 ^Ac^** | **35.66 ± 1.48 ^Ac^** | **41.54 ± 5.92 ^Ab^** |
| **EB20** | **141.00 ± 8.71^Be^** | **158.00 ± 15.52 ^Be^** | **261 ± 36.70 ^Ae^** | **9.95 ± 0.49 ^Be^** | **10.26 ± 0.47 ^Be^** | **17.07 ± 2.78 ^Ad.^** |
| **W** | **140.66 ± 5.85 ^Ce^** | **157.33 ± 7.02 ^Be^** | **263.44 ± 2.97 ^Ae^** | **9.74 ± 0.55 ^Be^** | **10.63 ± 0.47  ^Be^** | **17.50 ± 0.24 ^Ad^** |

*Small shared letters indicate no significant differences in each column and large shared letters indicate no significant difference in each row at 95% confidence level.

| **Overall accpetance** | | | |
| --- | --- | --- | --- |
| **Sample** | **First day** | **Third day** | **Fifth day** |
| **BS30** | **3.43±0.75 ^Abc*^** | **2.33 ± 0.28 ^Ab^** | **2.33 ± 0.78 ^Ab^** |
| **BB30** | **2.43 ± 0.51 ^Acd^** | **2 ± 0.5 ^Ab^** | **1.83 ± 0.28 ^Ab^** |
| **BB20** | **4.22±0.57 ^Aab^** | **3.83 ± 0.76 ^Aa^** | **3.66 ± 0.19 ^Aa^** |
| **BS20** | **4.44± 0.5 ^Aab^** | **4 ± 0.1 ^Aa^** | **4 ±0.0 ^Aa^** |
| **ES30** | **2.22± 0.69 ^Ad^** | **2 ± 0.76 ^Ab^** | **2.33 ± 0.57 ^Ab^** |
| **ES20** | **3.66 ± 0.76 ^Aab^** | **3.55 ± 0.5 ^Aa^** | **3.33 ± 1.04 ^Aa^** |
| **EB30** | **2.55 ± 0.51 ^Acd^** | **2.5 ± 0.5 ^Ab^** | **2.16 ± 0.28 ^Ab^** |
| **EB20** | **4.66 ± 0.57 ^Aa^** | **4.16 ±0.76 ^Aa^** | **4.16 ± 0.28 ^Aa^** |
| **W** | **4.5 ± 0.5 ^Aab^** | **4.33 ± 0.28 ^Aa^** | **4 ± 0.1 ^Aa^** |

Table 2 Effect of extrusion parameters, amount and particle size of the soybean hull fractions on the overall accptance during storage (mean ± standard deviation)

**Fig.1** TPA graph of EB20 on the first day after production

**Fig.2**: DSC thermograms of EB20 on the first day after production


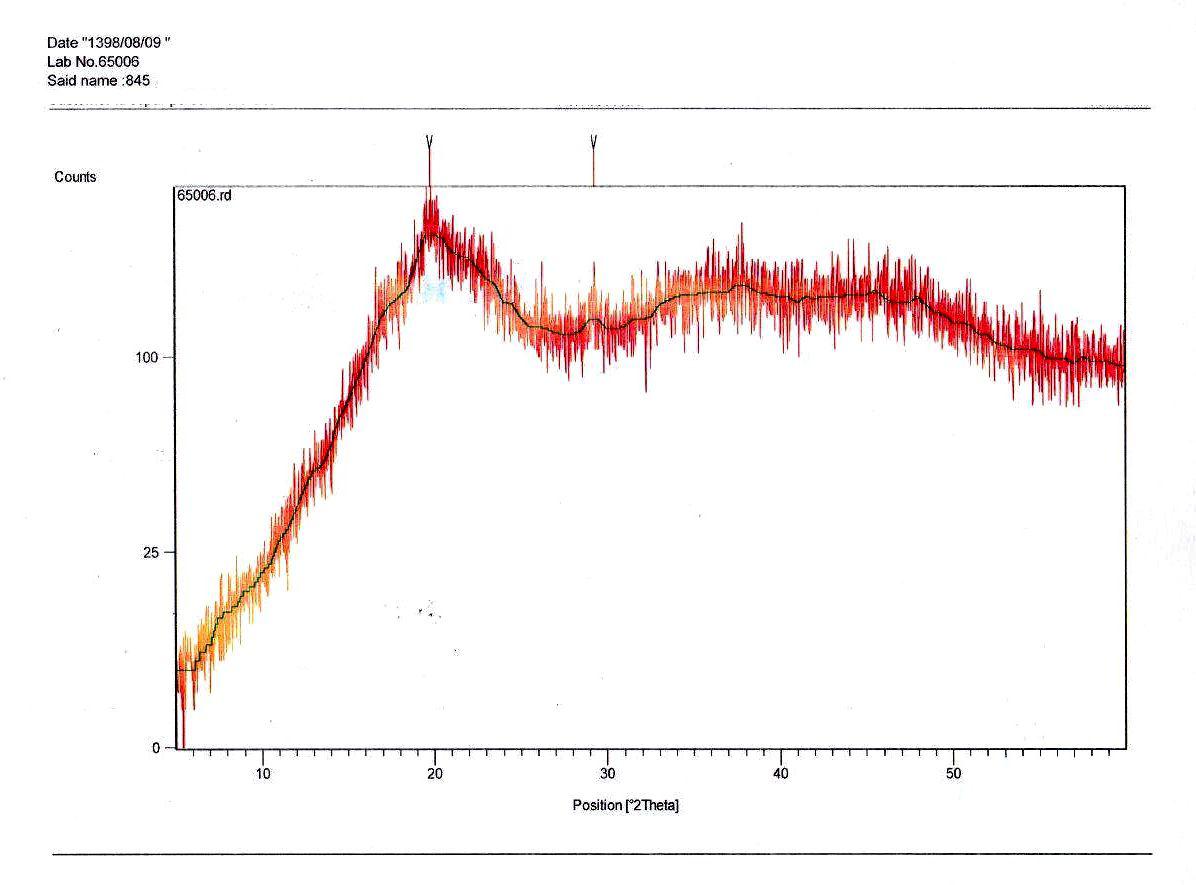


**Fig.3** XRD diffractogram of EB20 on the first day after production


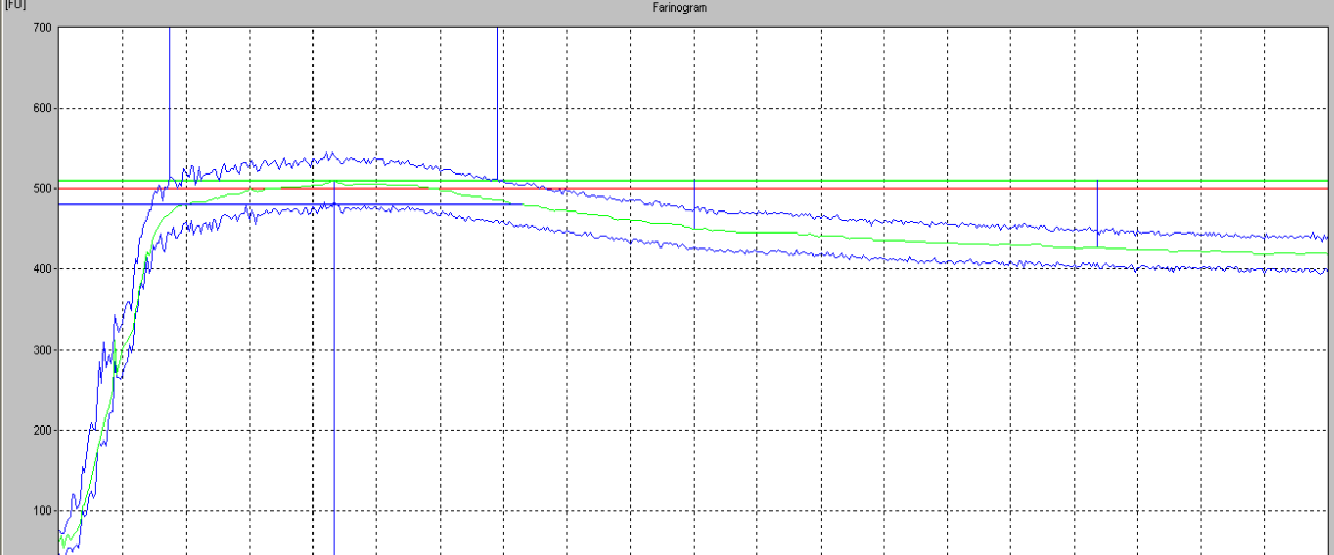


**Fig.4** Farinogram blend of EB20 and wheat flour

**Fig.5** Particle size distribution of ES


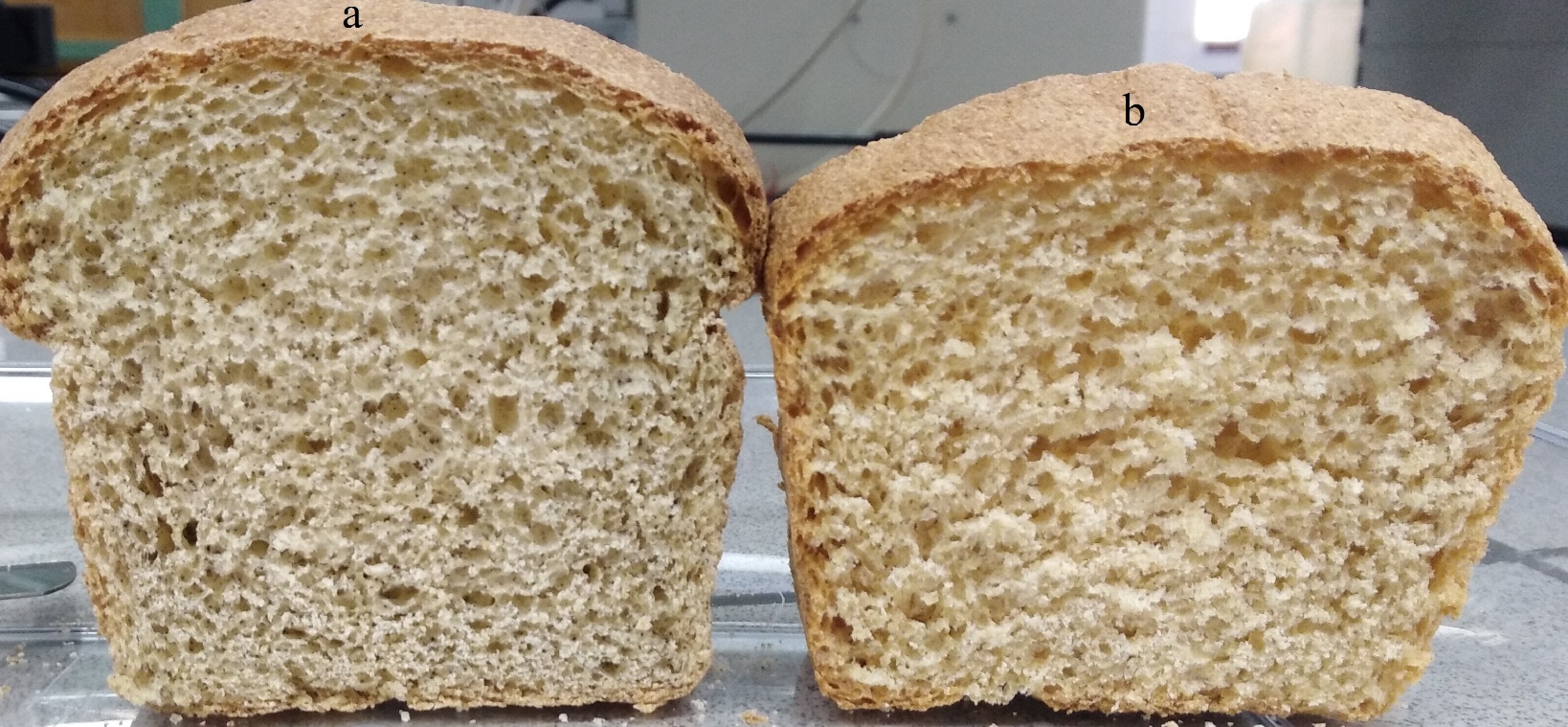


**Fig.6** Cross-sectional image of EB20 (a) and W (b) on the third day after production
